# Clinical significance of lactate clearance in patients with cardiogenic shock: results from the RESCUE registry

**DOI:** 10.1186/s40560-021-00571-7

**Published:** 2021-10-18

**Authors:** Ik Hyun Park, Jeong Hoon Yang, Woo Jin Jang, Woo Jung Chun, Ju Hyeon Oh, Yong Hwan Park, Young-Guk Ko, Cheol Woong Yu, Bum Sung Kim, Hyun-Joong Kim, Hyun Jong Lee, Jin-Ok Jeong, Hyeon-Cheol Gwon

**Affiliations:** 1 Division of Cardiology, Samsung Changwon Hospital, Sungkyunkwan University School of Medicine, Changwon, Republic of Korea; 2 Division of Cardiology, Heart Vascular Stroke Institute, Samsung Medical Center, Sungkyunkwan University School of Medicine, Seoul, Republic of Korea; 3 Division of Cardiology, Department of Internal Medicine, Seoul Hospital, Ewha Womans University College of Medicine, 260, Gonghang-daero, Gangseo-gu, 07804 Seoul, Republic of Korea; 4 Division of Cardiology, Severance Cardiovascular Hospital, Yonsei University College of Medicine, Seoul, Republic of Korea; 5 Division of Cardiology, Korea University Anam Hospital, Seoul, Republic of Korea; 6 Division of Cardiology, Konkuk University Medical Center, Seoul, Republic of Korea; 7 Division of Cardiology, Sejong General Hospital, Bucheon, Republic of Korea; 8 Division of Cardiology, Chungnam National University Hospital, Daejeon, Republic of Korea

**Keywords:** Cardiogenic shock, Lactate clearance, Prognosis

## Abstract

**Background:**

Limited data are available on the clinical significance of lactate clearance (LC) in patients with cardiogenic shock (CS). This study investigated the prognostic role of LC in CS patients.

**Methods:**

We analyzed data from 628 patients in the RESCUE registry, a multicenter, observational cohort enrolled between January 2014 and December 2018. Univariable logistic regression analysis was performed to determine the prognostic implications of 24 h LC, and then patients were divided into two groups according to the cut-off value of 24 h LC (high lactate clearance [HLC] group vs. low lactate clearance [LLC] group). The primary outcome was in-hospital mortality. We also assessed all-cause mortality at 12 month follow-up and compared the prognostic performance of 24 h LC according to initial serum lactate level.

**Results:**

In the univariable logistic regression analysis, 24 h LC was associated with in-hospital mortality (odds ratio 0.989, 95% confidence interval [CI] 0.985–0.993, *p* < 0.001), and the cut-off value for the LC of the study population was 64%. The HLC group (initial 24 h LC ≥ 64%, n = 333) had a significantly lower incidence of in-hospital death than the LLC group (*n* = 295) (25.5% in the HLC group vs. 42.7% in the LLC group, *p* < 0.001). During 12 months of follow-up, the cumulative incidence of all-cause death was significantly lower in the HLC group than in the LLC group (33.0% vs. 48.8%; hazard ratio 0.55; 95% CI 0.42–0.70; *p* < 0.001). In subgroup analysis, 24 h LC predicted in-hospital mortality better in patients with initial serum lactate > 5 mmol/L than in those with serum lactate ≤ 5 mmol/L (c-statistics of initial serum lactate > 5 mmol/L = 0.782 vs. c-statistics of initial serum lactate ≤ 5 mmol/L = 0.660, *p* = 0.011).

**Conclusions:**

Higher LC during the early phase of CS was associated with reduced risk of in-hospital and 12 month all-cause mortalities. Patients with LC ≥ 64% during the 24 h after CS onset could expect a favorable prognosis, especially those with an initial serum lactate > 5 mmol/L.

*Trial registration:* RESCUE (REtrospective and prospective observational Study to investigate Clinical oUtcomes and Efficacy of left ventricular assist device for Korean patients with cardiogenic shock), NCT02985008, Registered December 5, 2016—retrospectively and prospectively registered, https://clinicaltrials.gov/ct2/show/record/NCT02985008

**Supplementary Information:**

The online version contains supplementary material available at 10.1186/s40560-021-00571-7.

## Background

Lactate has been studied over time in patients with shock, and serum lactate is an important prognostic factor that reflects decreased oxygen delivery and tissue hypoperfusion [1, 2]. Previous studies have suggested that serum lactate clearance could be a clinically reliable surrogate for the magnitude and duration of tissue hypoxia in shock patients and demonstrated the prognostic value of lactate clearance measured at 8–48 h after initiation of shock treatment in terms of clinical outcomes [2–4]. To date, the prognostic role of lactate clearance in patients with septic shock has been reported in many studies [5, 6], including a randomized clinical trial in which lactate clearance-guided therapy was not inferior to central venous oxygen saturation-guided therapy in patients with septic shock [7]. In contrast, limited data are available on the clinical significance of lactate clearance in cardiogenic shock (CS). A small pilot study reported that 12 h lactate clearance < 10% was associated with significantly lower survival in CS following ST-segment elevation myocardial infarction [8]. Another study reported that a single lactate value and lactate clearance measured at 24 h rather than baseline lactate level were predictive for 30 day mortality in CS patients undergoing extracorporeal membrane oxygenation (ECMO) [2]. Therefore, serial measurements of lactate are recommended during shock treatment to evaluate hemodynamic status and optimize therapy [4, 9]. Only one study has reported that lactate clearance is a better predictive marker than baseline lactate in CS [8], and other studies only analyzed specific populations of CS patients who were supported with ECMO. In these studies, the cut-off values for lactate clearance varied from 10 to 70% and were not useful in most CS patients [2, 3, 8]. Furthermore, a recent study showed that lactate measured at 8 h after shock onset had greater predictive value than the 8-h lactate clearance in CS patients [10]. For these reasons, the optimal clearance value and appropriate time point for measuring lactate clearance as a prognostic marker remain controversial. We evaluated the association between 24 h lactate clearance and clinical outcomes and compared the prognostic role of 24 h lactate clearance according to initial serum lactate level in patients with CS.

## Methods

### Study design and patients

**Fig. 1:**
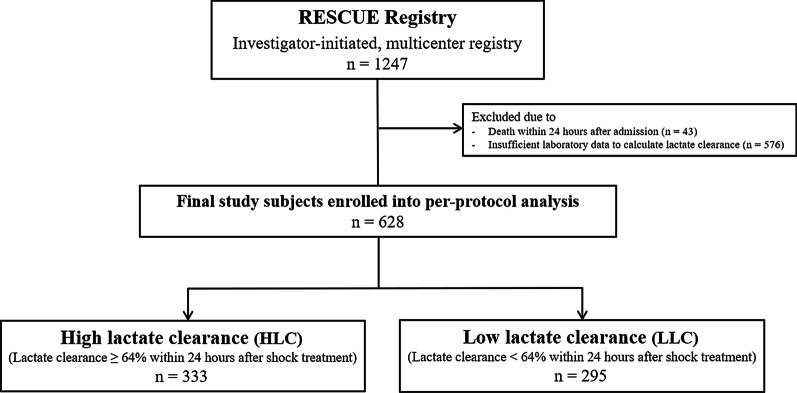
Schematic illustration of study cohort selection

The RESCUE (REtrospective and prospective observational Study to investigate Clinical outcomes and Efficacy of left ventricular assist device for Korean patients with cardiogenic shock; NCT02985008 at www.clinicaltrials.gov) study is a multicenter, retrospective and prospective registry of patients with CS. Between January 2014 and December 2018, 1,247 consecutive patients with CS (954 enrolled retrospectively and 293 enrolled prospectively) were recruited from 12 tertiary centers in the Republic of Korea. More detailed information about prospective and retrospective enrollment at each institute is shown in Additional file 1: Table S1. The inclusion criteria were as follows: (1) age ≥ 19 years, (2) systolic blood pressure < 90 mmHg for 30 min or need for inotrope or vasopressor support to achieve systolic blood pressure > 90 mmHg, and (3) presence of pulmonary congestion and signs of impaired organ perfusion (altered mental status, cold periphery, oliguria < 0.5 mL/kg/hour for the previous six hours, or blood lactate > 2.0 mmol/L) [11]. Major exclusion criteria were out-of-hospital cardiac arrest, other causes of shock (hypovolemic or septic shock), and refusal of active treatment [12]. For the present study, we excluded those who died within 24 h of admission and those for whom serial lactate level was unavailable. Finally, 628 patients were divided into two groups according to 24 h lactate clearance ≥ 64% (the cut-off value of the study population, determined 24 h after initiation of shock treatment) or not (Fig. 1). The study protocol was approved by the institutional review board (IRB) of each hospital, and the study was conducted according to the principals of the Declaration of Helsinki. The IRBs of the participating hospitals waived the requirement for informed consent for retrospectively enrolled patients, and all prospectively enrolled patients provided written informed consent before enrollment.

### Data collection and study outcomes

For the RESCUE registry, information about patient demographics, in-hospital management, laboratory data, procedural data, and outcomes was collected by independent clinical research coordinators via web-based case report forms. Additional information was obtained by further inquiry into medical records or telephone contact, if necessary [12]. Laboratory findings, such as serum creatinine and hemoglobin, exhibiting the worst values in the 24 h after initiation of shock treatment were collected. The primary outcome was in-hospital mortality, and the secondary outcome was all-cause mortality during 12 months of follow-up. We also analyzed the prognostic performance of 24 h lactate clearance according to initial serum lactate level > 5 mmol/L or not [13].

### Lactate measurement and lactate clearance

Serum lactate values were measured serially using arterial blood gas analysis in intensive care units from the beginning of shock treatment until hemodynamically stable conditions were achieved. After that, they were measured every 24 h. Previous studies have used various time points for lactate clearance, from 8 to 24 h. We chose 24 h lactate clearance for our analysis because baseline and 24 h lactate values were available in our registry, and that time point has been suggested as a good predictor of mortality in patients with shock [3, 14]. We calculated 24 h lactate clearance using the following equation: 24 h lactate clearance = (initial lactate value—lactate value 24 h after the initial time point)/initial lactate value [2, 9]. The prognostic role of 24 h lactate clearance in CS patients was investigated using a univariable logistic regression model. After it was confirmed that 24 h lactate clearance was associated with in-hospital mortality, the cut-off value for lactate clearance was used as a threshold to separate the groups.

### Statistical analysis

Categorical variables are presented as the counts and percentages and were compared using the χ^2^ test or Fisher’s exact test, as appropriate. Continuous variables were compared with Student’s *t*-test or Mann–Whitney *U*-test and are presented as medians (interquartile ranges, 25–75th percentile) for variables lacking a normal distribution. Cumulative event rates were estimated with the Kaplan–Meier method and compared with log-rank tests. The best discriminative values were obtained from the receiver operating characteristic (ROC) curve using ROC01, a method that minimizes the distance between the ROC plot and the point (0, 1). The area under the receiver operating characteristic (AUROC) was calculated to quantify the accuracy of lactate clearance in predicting in-hospital mortality, and Delong's test for two correlated ROC curves was performed for comparison of the area under the curve (AUC) [15]. Univariable and multivariable logistic regression models were used to predict in-hospital mortality. Covariates that were either statistically significant on univariable analysis (*p* value < 0.1) or considered clinically important were included in multivariable models. Analyzed covariates were lactate clearance, age, sex, body mass index, diabetes mellitus, hypertension, dyslipidemia, current smoking, chronic kidney disease, ischemic cardiomyopathy, and serum glucose. All probability values were two-tailed, and *p* values < 0.05 were considered statistically significant. Statistical analyses were performed using SPSS software, version 23 (IBM, Armonk, NY, USA).

## Results

### Baseline characteristics

We analyzed 628 patients, and upon using univariable logistic regression analysis to determine the prognostic implications of lactate clearance in CS, a significant association between 24 h lactate clearance and in-hospital mortality was observed (odds ratio [OR] 0.989, 95% CI 0.985–0.993, *p* < 0.001). The cut-off value for lactate clearance of our study population was 64% that was obtained from the ROC curve using the ROC01 method, and the study population was divided into those with lactate clearance ≥ 64% (high lactate clearance [HLC] group, n = 333) and those with lactate clearance < 64% (low lactate clearance [LLC] group, n = 295). The baseline clinical characteristics of the study population are shown in Table 1. The median age of the total study population was 66 years, and 416 patients (66.2%) were men. There were no significant differences in demographics or co-morbidities between the HLC group and the LLC group. About half of the study population suffered from hypertension, and more than one-quarter of the study population suffered from diabetes mellitus or dyslipidemia. Ischemic cardiomyopathy was the most common cause of CS, and its incidence was similar in the two groups (71.2% in the HLC group vs. 71.9% in the LLC group). Left ventricular ejection fraction (LVEF) at admission and lowest LVEF during follow-up were similar between the two groups (*p* = 0.661 and *p* = 0.405, respectively). There were no significant differences in systolic and diastolic blood pressure (*p* = 0.070 and *p* = 0.104), or heart rate (*p* = 0.979) between the two groups.

**Table 1:** Baseline clinical characteristics

	High lactate clearance	Low lactate clearance	*p* value
	*n* = 333	*n* = 295	
Age	65.0 (55.0–75.0)	68.0 (56.0–78.0)	0.096
Male	227 (68.2)	189 (64.1)	0.278
Body mass index (kg/m^2^)	23.0 (20.8–25.7)	22.9 (20.4–25.7)	0.900
Diabetes mellitus	115 (34.5)	104 (35.3)	0.850
Hypertension	161 (48.3)	151 (51.2)	0.478
Dyslipidemia	87 (26.1)	76 (25.8)	0.917
Current smoker	94 (28.2)	70 (23.7)	0.200
Chronic kidney disease	35 (10.5)	38 (12.9)	0.355
Peripheral vascular disease	19 (5.7)	11 (3.7)	0.246
Previous myocardial infarction	43 (12.9)	37 (12.5)	0.889
Previous percutaneous coronary intervention	48 (14.4)	42 (14.2)	0.950
Previous coronary artery bypass grafting	11 (3.3)	7 (2.4)	0.486
Previous cerebrovascular accident	41 (12.3)	27 (9.2)	0.203
Clinical presentation			0.010
Ischemic cardiomyopathy	237 (71.2)	212 (71.9)	
Non–ischemic cardiomyopathy	54 (16.2)	55 (18.6)	
Pulmonary thromboembolism	13 (3.9)	6 (2.0)	
Refractory arrhythmia	20 (6.0)	5 (1.7)	
Other causes	9 (2.7)	17 (5.8)	
Echocardiographic findings			
Left ventricular ejection fraction at admission (%)	33.0 (25.0–50.9)	33.0 (24.8–44.6)	0.661
Lowest left ventricular ejection fraction (%)	30.0 (20.0–46.0)	30.0 (20.5–39.9)	0.405
Systolic blood pressure (mmHg)	70.0 (59.0–80.0)	72.0 (60.0–82.0)	0.070
Diastolic blood pressure (mmHg)	46.0 (35.0–54.0)	46.0 (40.0–56.0)	0.104
Heart rate (beat/minute)	89.0 (62.0–111.0)	90.0 (64.3–108.8)	0.979

Values are shown as *n* (%) or median (interquartile range)

### Shock treatment and laboratory characteristics

Laboratory and shock treatment characteristics according to lactate clearance are shown in Table 2. The median value of lactate clearance of the study population was 66.0% (interquartile range 38.9–80.7). Lactate clearance of the HLC group and the LLC group were 80.0% and 36.2%, respectively. The initial blood levels of hemoglobin (mg/dL), glucose (mg/dL), and lactate (mmol/L) were significantly higher in the HLC group than in the LLC group (*p* = 0.039, *p* = 0.001 and *p* < 0.001, respectively). Vasoactive inotropic score was lower in the HLC group than in the LLC group (*p* = 0.006). Mechanical circulatory support and insertion of central venous line were performed similarly between the two groups (*p* = 0.134 and *p* = 0.537, respectively). The rate of ECMO application during the first 24 h of the lactate measurement period was lower in the HLC group, but the difference was not significant (*p* = 0.066). The median value of shock-to-ECMO time in patients who underwent ECMO within the first 24 h was similar between the two groups (66 min vs. 77 min, *p* = 0.267). Extracorporeal cardiopulmonary resuscitation was performed less frequently in the HLC group than in the LLC group, but the difference was not significant (*p* = 0.077). The HLC group underwent continuous renal replacement therapy less frequently than the LLC group (*p* < 0.001). The length of intensive care unit stay and hospital stay were significantly longer in the HLC group than in the LLC group (*p* = 0.002 and *p* = 0.001, respectively).

**Table 2:** Laboratory and shock treatment characteristics

	High lactate clearance	Low lactate clearance	*p* value
	*n* = 333	*n* = 295	
Laboratory findings, Initial			
Hemoglobin (mg/dL)	12.7 (10.5–14.7)	12.2 (10.5–14.0)	0.039
Serum glucose (mg/dL)	222.0 (150.0–319.0)	183.0 (134.0–269.0)	0.001
Total bilirubin (mg/dL)	0.6 (0.4–1.2)	0.7 (0.5–1.2)	0.021
Creatinine (mg/dL)	1.3 (1.0–1.7)	1.2 (0.9–1.8)	0.085
Lactate (mmol/L, at admission)	6.9 (4.7–1.0)	3.7 (2.3–7.1)	< 0.001
Lactate (mmol/L, 24 h after admission)	1.2 (0.9–1.7)	2.3 (1.4–6.8)	< 0.001
Lactate clearance (%)	80.0 (72.1–86.5)	36.2 (0.0–53.6)	< 0.001
NT–proBNP (pg/mL)	5884.5 (1015.5–21,827.8)	5152.0 (1506.5–13,628.5)	0.424
Vasoactive inotropic score	27.7 (8.0–80.0)	37.0 (10.0–110.0)	0.006
Use of venous central line	218 (65.5)	200 (67.8)	0.537
Use of mechanical circulatory support			0.134
None	138 (41.4)	97 (32.9)	
ECMO	126 (37.8)	127 (43.1)	
IABP	51 (15.3)	48 (16.3)	
ECMO + IABP	18 (5.4)	23 (7.8)	
Shock-to-ECMO time < 24 h	126 (37.8)	133 (45.1)	0.066
ECPR	62 (18.6)	72 (24.4)	0.077
Continuous renal replacement therapy	75 (22.5)	109 (36.9)	< 0.001
Mechanical ventilation	219 (65.8)	198 (67.1)	0.720
Length of intensive care unit stay (day)	8.0 (4.0–16.5)	6.0 (2.0–15.0)	0.002
Length of hospital stay (day)	14.0 (8.0–28.0)	11.0 (5.0–27.0)	0.001

Values are shown as *n* (%) or median (interquartile range)

*ECMO* extracorporeal membrane oxygenation, *ECPR* extracorporeal cardiopulmonary resuscitation, *IABP* intra-aortic balloon pump, *Nt-proBNP* N-terminal pro B-type natriuretic peptide

### Clinical outcomes

The rate of in-hospital mortality was lower in the HLC group than in the LLC group (25.5% vs. 42.7%, *p* < 0.001). In-hospital cardiac mortality was observed less frequently in the HLC group than in the LLC group (16.5% vs. 38.3%, *p* < 0.001). There were no differences in the incidence of stroke (2.7% vs. 1.4%, *p* = 0.237), gastrointestinal bleeding (2.4% vs. 3.4%, *p* = 0.459), or sepsis (3.3% vs. 1.4%, *p* = 0.111) during hospitalization between the two groups (Table 3).

**Table 3:** In-hospital clinical outcomes

	High lactate clearance	Low lactate clearance	*p* value
	*n* = 333	*n* = 295	
All-cause death	85 (25.5)	126 (42.7)	< 0.001
Cardiac death	55 (16.5)	113 (38.3)	< 0.001
Stroke	9 (2.7)	4 (1.4)	0.237
Gastrointestinal bleeding	8 (2.4)	10 (3.4)	0.459
Sepsis	11 (3.3)	4 (1.4)	0.111

Values are shown as *n* (%)

**Fig. 2:**
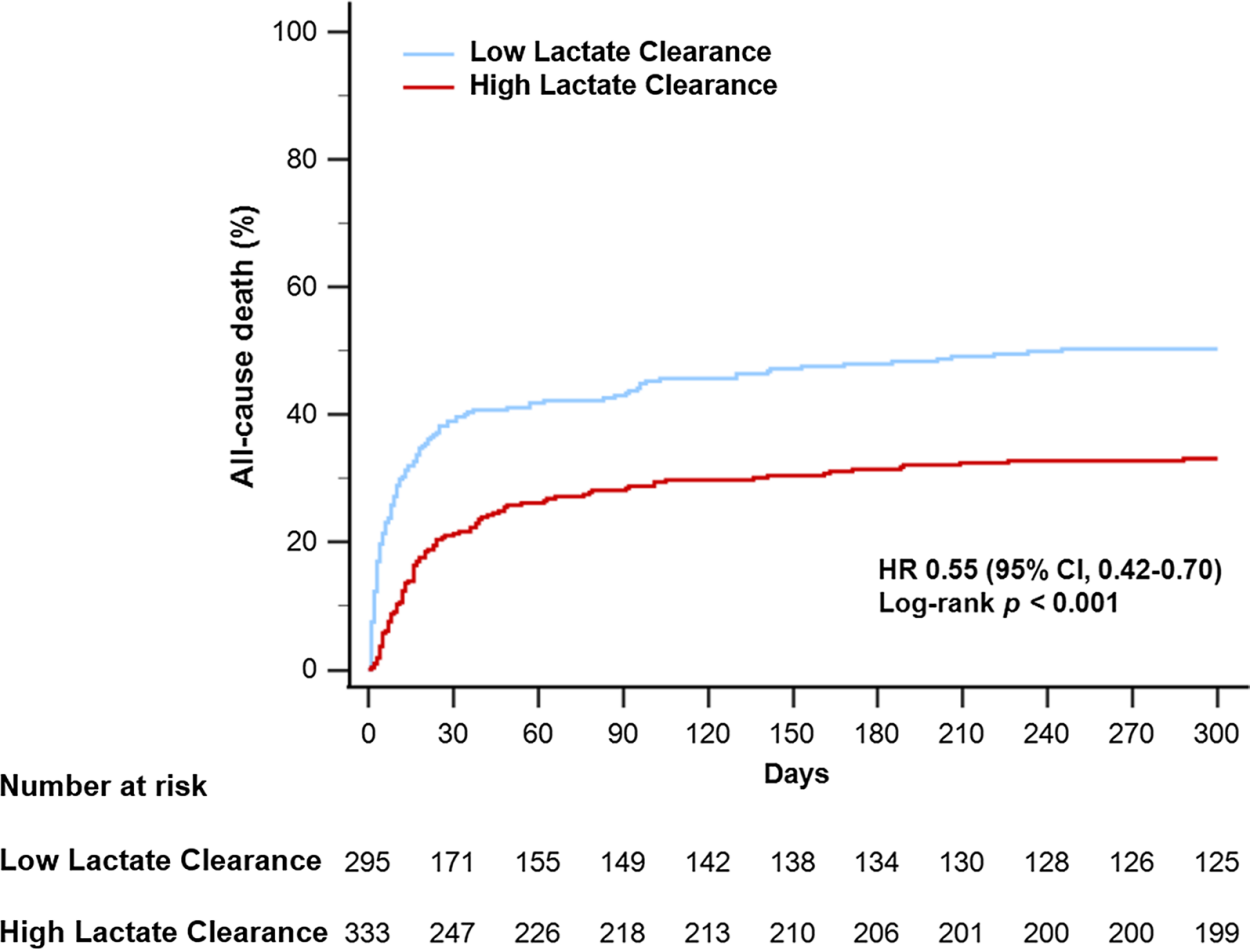
The cumulative incidence of all-cause mortality during 12 month follow-up according to lactate clearance. Kaplan–Meier curves are presented to compare the cumulative incidence of all-cause mortality between the low lactate clearance group and the high lactate clearance group. *CI* confidence interval, *HR* hazard ratio

During follow-up, the rates of all-cause mortality (33.0% in the HLC group vs. 48.8% in the LLC group, hazard ratio [HR] 0.55, 95% confidence interval [CI] 0.42–0.70, *p* < 0.001) (Fig. 2) and cardiac death (24.3% vs. 43.7%, HR 0.45, 95% CI 0.34–0.60, *p* < 0.001) were significantly lower in the HLC group than in the LLC group. The incidence of myocardial infarction (1.2% vs. 0.7%, HR 1.38, 95% CI 0.27–7.04, *p* = 0.700) and cerebrovascular accident (0.9% vs. 1.4%, HR 0.50, 95% CI 0.11–2.27, *p* = 0.371) did not differ significantly between the two groups, but the rate of heart failure readmission tended to be higher in the HLC group than in the LLC group (8.4% vs. 3.7%, HR 1.65, 95% CI 0.83–3.13, *p* = 0.124) (Additional file 1: Tables S2 and S3).

### Prognostic factors of in-hospital mortality

In multivariable logistic regression analysis, independent predictors of in-hospital mortality included 24 h lactate clearance ≥ 64% (OR 0.42, 95% CI 0.30–0.60, *p* < 0.001), chronic kidney disease (OR 1.95, 95% CI 1.17–3.26, *p* = 0.011), ischemic cardiomyopathy (OR 1.52, 95% CI 1.01–2.31, *p* = 0.046), and baseline serum glucose above the median value of 203 mg/dL (OR 1.45, 95% CI 1.01–2.08, *p* = 0.046) (Additional file 2: Figure S1).

### Association of lactate clearance with clinical outcomes

**Fig. 3:**
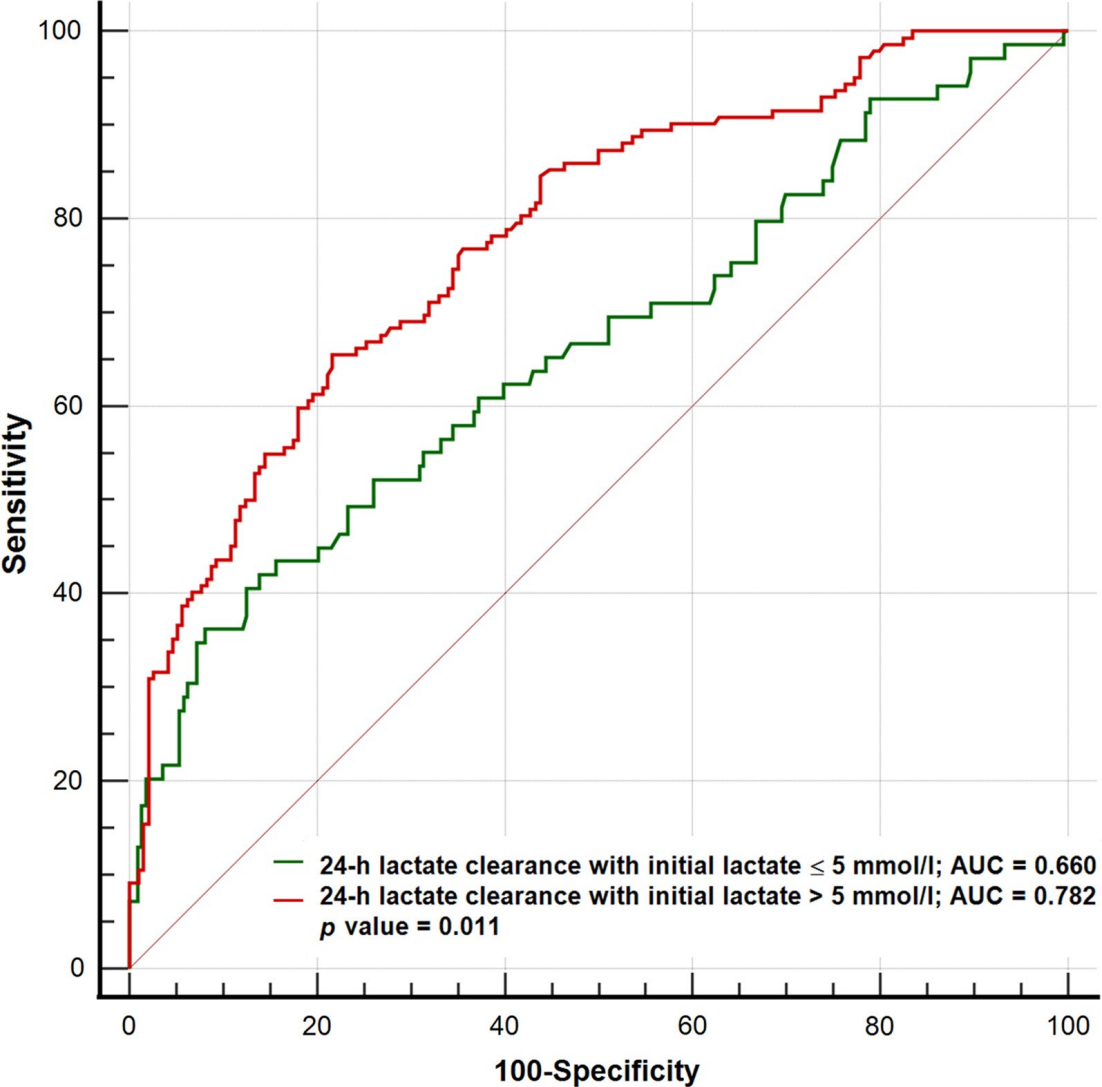
ROC curves of lactate clearance to predict in-hospital mortality according to initial serum lactate level. ROC curves show the comparison of 24 h lactate clearance to predict in-hospital mortality according to initial serum lactate level > 5 mmol/L or not. *AUC* area under the curve, *ROC* receiver operating characteristic

We performed a subgroup analysis to investigate the relationship between in-hospital mortality and serum lactate clearance in the first 24 h after initiation of shock treatment according to baseline serum lactate level. The study population was divided into two groups according to baseline serum lactate level > 5 mmol/L or not (initial serum lactate > 5 mmol/L vs. initial serum lactate ≤ 5 mmol/L), and we calculated and compared the AUROC of 24 h lactate clearance to predict the in-hospital mortality of each group. In that analysis, predictive performance of 24 h lactate clearance for in-hospital mortality was significantly higher in patients with initial serum lactate > 5 mmol/L than in those with initial serum lactate ≤ 5 mmol/L (0.782 vs. 0.660, *p* = 0.011) (Fig. 3). Subgroup analysis was performed to compare AUROC of 24 h lactate clearance to predict in-hospital mortality according to mechanical circulatory support within the first 24 h of the lactate clearance measurement period. The AUC of the overall population was 0.668 and, the IABP or medical therapy alone subgroup showed a higher AUC than the ECMO subgroup, but the difference was not significant (AUC of IABP or medical therapy alone = 0.684 vs. AUC of ECMO = 0.640, respectively; *p* = 0.344) (Additional file 3: Figure S2).

## Discussion

We investigated the association between 24 h serum lactate clearance after initiation of shock treatment and clinical outcomes in patients with CS. The main finding of our study is that higher 24 h lactate clearance was associated with reduced risk of in-hospital and 12 month all-cause mortalities compared with lower 24 h lactate clearance in patients with CS. In particular, the prognostic role of 24 h lactate clearance was prominent in severe forms of CS with high initial serum lactate, suggesting that 24 h lactate clearance could be a treatment goal in refractory CS. To the best of our knowledge, this is the first study to examine the clinical effects of serum lactate clearance on the prognosis of diverse cardiac diseases that present with CS according to baseline serum lactate levels. Our findings correspond well with those of earlier studies that established an association between poor lactate clearance and adverse clinical outcomes.

Serum lactate varies in proportion to ongoing tissue hypoxia, and reduction of lactate level is a marker of successful resuscitation because lactate clearance indicates restored oxygen delivery [7]. Previous studies analyzing the association between lactate clearance and clinical outcome were performed mostly in patients with sepsis, and they reported that early lactate clearance above 10% was an important determinant of survival [5, 16]. Regarding the prognostic role of lactate clearance in CS, Attanà et al. reported that 12 h lactate clearance < 10% identified a subset of patients at higher risk for death [8], and Slottosch et al. showed that lactate clearance across 24 h was predictive of 30 day mortality with a cut-off value of 68.7% in CS patients, concluding that the dynamic course of lactate is a valuable tool for predicting mortality. However, they analyzed only patients undergoing ECMO [2]. We evaluated the prognostic role of serum lactate clearance in clinical outcomes among CS patients using a large, multicenter, dedicated shock registry covering diverse cardiac diseases, and we identified 24 h lactate clearance < 64% as an indicator of higher risk for in-hospital mortality. Our threshold for lactate clearance (24 h lactate clearance ≥ 64%) could be a reliable surrogate marker for assessing successful therapy in a comprehensive cohort of CS patients, which could be used as an indicator in initial shock management.

Hyperlactatemia can be caused by hyperglycemia, catecholamines, or tissue hypoxia [17]. In our results, significant differences in vasoactive inotropic score and serum glucose were observed. These differences could be related to the initial higher lactate level in each group, and in the setting of comparing single lactate values, these variables could be confounding factors. Therefore, lactate-related criteria used to assess prognosis in CS should reflect the dynamic course of lactate levels over time [2, 8, 10]. Considering a previous study reporting that lactate clearance could not be influenced by the conditions accompanying shock [18], serum lactate clearance, which is calculated from two time points, reflects the time course of lactate level, and could show better predictive power for clinical outcomes than single lactate values. However, in a recently published study, Lee et al. classified participants as early (< 0.9 h), intermediate (0.9 to 2.2 h), and late (> 2.2 h) according to shock-to-ECMO time, and analysis showed a 47% lower risk of 30 day mortality in the early group than in the late group, suggesting that sooner may be better [19]. On the other hand, previous studies investigating the use of mechanical circulatory support in CS reported hemodynamic improvement without mortality benefit and recommended against using active mechanical circulatory support in unselected patients due to potential complications [20, 21]. Based on these results, for risk stratification reflecting these issues, 5-stage CS classification proposed by the Society for Cardiovascular Angiography and Intervention (SCAI) [22] should be used first to discriminate patients in urgent need of ECMO, and then 24 h lactate clearance could be used as an indicator of high risk for adverse clinical outcomes in selective CS patients such as those in SCAI classification stage C or D. This could prevent patients in lower stages from shifting to higher stages through appropriate timing of early therapeutic intervention, since the prevalence of hemodynamic deterioration after 24 h increases with higher SCAI shock stages [23].

We performed additional analyses to identify subsets of patients whose lactate clearance showed better predictive power for clinical outcomes. In an analysis for developing the IABP-SHOCK II risk score, arterial blood lactate > 5 mmol/L at admission emerged as an independent predictor of 30 day mortality and was used as a key parameter of the risk prediction scoring system [13]. Since the significance of lactate clearance has not been determined according to severity of shock, we performed subgroup analysis to find a subset of CS patients whose lactate clearance exhibited better discrimination for assessing prognostic roles based on initial serum lactate level. We analyzed the accuracy of serum lactate clearance for predicting in-hospital mortality according to initial serum lactate > 5 mmol/L and found that 24 h lactate clearance was significantly more predictive in patients whose initial serum lactate level was greater than 5 mmol/L than it was in those whose lactate levels were less than 5 mmol/L, with even better predictive ability than the single lactate value at 24 h after shock treatment onset. Considering that serum lactate clearance was significantly enhanced in patient treated with ECMO [24], we performed additional subgroup analysis comparing the predictive ability of 24 h lactate clearance for in-hospital mortality based on application of mechanical circulatory support in CS. Among those who received IABP support or medical therapy alone, serum lactate clearance predicted prognosis better than it did among those who underwent ECMO, but significance was not observed. Based on these results, 24 h lactate clearance could identify a subset of CS patients at high risk for adverse clinical outcomes; in particular, CS patients with high initial serum lactate level may undergo frequent assessments of arterial lactate, at least hourly or more frequent point-of-care testing [22].

Acute renal failure is a common occurrence in cardiogenic shock requiring renal replacement therapy in patients with multiple organ dysfunction. Previous studies [25, 26] reported that lactic acidosis could be treated successfully with renal replacement therapy, such that the serum lactate level might not reflect tissue hypoxia in cardiogenic shock. However, another study evaluating the effects of renal replacement therapy on lactate removal showed that renal replacement therapy was responsible for < 3% of total lactate clearance in critically ill patients; therefore, serum lactate level remains a reliable marker of tissue hypoxia [27]. From these various points of view, although it would not be likely to affect our results significantly, differences in the use of renal replacement therapy between the two groups could be a confounder in our study.

### Study limitations

This study has several limitations. First, its design was non-randomized and observational, potentially affecting the results through selection bias and confounding factors. Most patients presented with an ischemic etiology, and patients with non-ischemic causes were heterogeneous and of limited sample size. Second, our registry did not include hemodynamic parameters, such as cardiac index or pulmonary capillary wedge pressure measured by a pulmonary arterial catheter. Third, removal of serum lactate could occur in the liver or kidney, so the presence of underlying disease in those organs or use of continuous renal replacement therapy could affect lactate clearance and thereby our results. Fourth, although we focused on the prognostic role of lactate clearance rather than single lactate values, significantly different serum glucose values and vasoactive inotropic scores between the two groups may have affected our results, since hyperlactatemia can be caused by hyperglycemia, or catecholamines. Finally, treatment for CS (including cardiac arrest), such as the type or amount of intravenous fluids, vasopressors, and inotropes administered and mechanical circulatory support implanted, were left to the physician’s discretion, although all coronary interventions were based on guidelines from the Korean Circulation Society.

## Conclusions

An initial 24 h serum lactate clearance ≥ 64% in CS patients was associated with lower in-hospital mortality, with a prominent association noted in those whose initial serum lactate level was > 5 mmol/L. A large-scale, randomized trial is needed to confirm these findings.

## Supplementary Information

**Additional file 1: Table S1.** Prospective and retrospective enrollments of each Institute. **Table S2.** 12 month follow-up outcomes. **Table S3.** Baseline characteristics of survivor and non-survivor.

**Additional file 2: Figure S1. **Predictors of in-hospital mortality.

**Additional file 3: Figure S2. **ROC curves of lactate clearance to predict in-hospital mortality according to the application of mechanical circulatory support.

## Data Availability

The data used during the current study are available from the corresponding author on reasonable request.

## Abbreviations

CS: Cardiogenic shock
ECMO: Extracorporeal membrane oxygenation
HLC: High lactate clearance
LLC: Low lactate clearance
LVEF: Left ventricular ejection fraction
ROC: Receiver operating characteristic
