# Supplementary material for: Clinical significance of lactate clearance in patients with cardiogenic shock: results from the RESCUE registry
Source: J Intensive Care. 2021 Oct 18;9:63. doi: 10.1186/s40560-021-00571-7 (PMC8522140; doi:10.1186/s40560-021-00571-7)
Supplement: Supplementary file 1 — Additional file 1: Table S1. Prospective and retrospective enrollments of each Institute. Table S2. 12 month follow-up outcomes. Table S3. Baseline characteristics of survivor and non-survivor. [file 40560_2021_571_MOESM1_ESM.doc]

**Supplemental Appendix**

**(1) Supplementary table**

**(2) Supplementary figure legend**

**Supplementary Table 1. Prospective and Retrospective Enrollments of each Institute**

| **Institutes** | **Overall population**  **n = 1,247** | **Retrospective**  **n = 954** | **Prospective**  **n = 293** |
| --- | --- | --- | --- |
| **Samsung Medical Center** | 249 | 144 | 105 |
| **Severance Cardiovascular Hospital** | 181 | 147 | 34 |
| **Korea University Anam Hospital** | 134 | 130 | 4 |
| **Samsung Changwon Hospital** | 122 | 46 | 76 |
| **Konkuk University Hospital** | 112 | 89 | 23 |
| **Chungbuk National University Hospital** | 91 | 90 | 1 |
| **Inje University Ilsan Paik Hospital** | 78 | 64 | 14 |
| **Sejong General Hospital** | 66 | 60 | 6 |
| **Chung-Ang University Hospital** | 67 | 63 | 4 |
| **Chungnam National University Hospital** | 57 | 57 | 0 |
| **Inha University Hospital** | 52 | 32 | 20 |
| **Dankook University Hospital** | 38 | 32 | 6 |

**Supplementary Table 2. 12-month follow-up Outcomes**

|  | **High lactate clearance** | **Low lactate clearance** | **HR** | ***p* value** |
| --- | --- | --- | --- | --- |
|  | **n = 333** | **n = 295** | **95% CI** |  |
| **All-cause death** | 110 (33.0) | 144 (48.8) | 0.55 (0.42 – 0.70) | <0.001 |
| **Cardiac death** | 81 (24.3) | 129 (43.7) | 0.45 (0.34 – 0.60) | <0.001 |
| **Myocardial infarction** | 4 (1.2) | 2 (0.7) | 1.38 (0.27 – 7.04) | 0.700 |
| **Cerebrovascular accident** | 3 (0.9) | 4 (1.4) | 0.50 (0.11 – 2.27) | 0.371 |
| **Heart failure readmission** | 28 (8.4) | 11 (3.7) | 1.65 (0.87 – 3.13) | 0.124 |
| Values are n (%). CI = confidence interval; HR = hazard ratio. | | | | |

**Supplementary Figure 1. Predictors of in-hospital mortality**

Forest plots show the results of multivariable analysis of predictors of in-hospital mortality in cardiogenic shock.

CI = confidence interval.

**Supplementary Figure 2. ROC curves of lactate clearance to predict in-hospital mortality according to the application of mechanical circulatory support**

ROC curves show the comparison of 24-hour lactate clearance to predict in-hospital mortality according to mechanical circulatory support.

AUC = area under curve, ECMO = extracorporeal membrane oxygenation, IABP = intra-aortic balloon pump, ROC = receiver operating characteristic.
