# Supplementary material for: Clinical significance of lactate clearance in patients with cardiogenic shock: results from the RESCUE registry
Source: J Intensive Care. 2021 Oct 18;9:63. doi: 10.1186/s40560-021-00571-7 (PMC8522140; doi:10.1186/s40560-021-00571-7)

**Additional file 2: Figure S1. Predictors of in-hospital mortality**

Forest plots show the results of multivariable analysis of predictors of in-hospital mortality in cardiogenic shock.

CI = confidence interval.


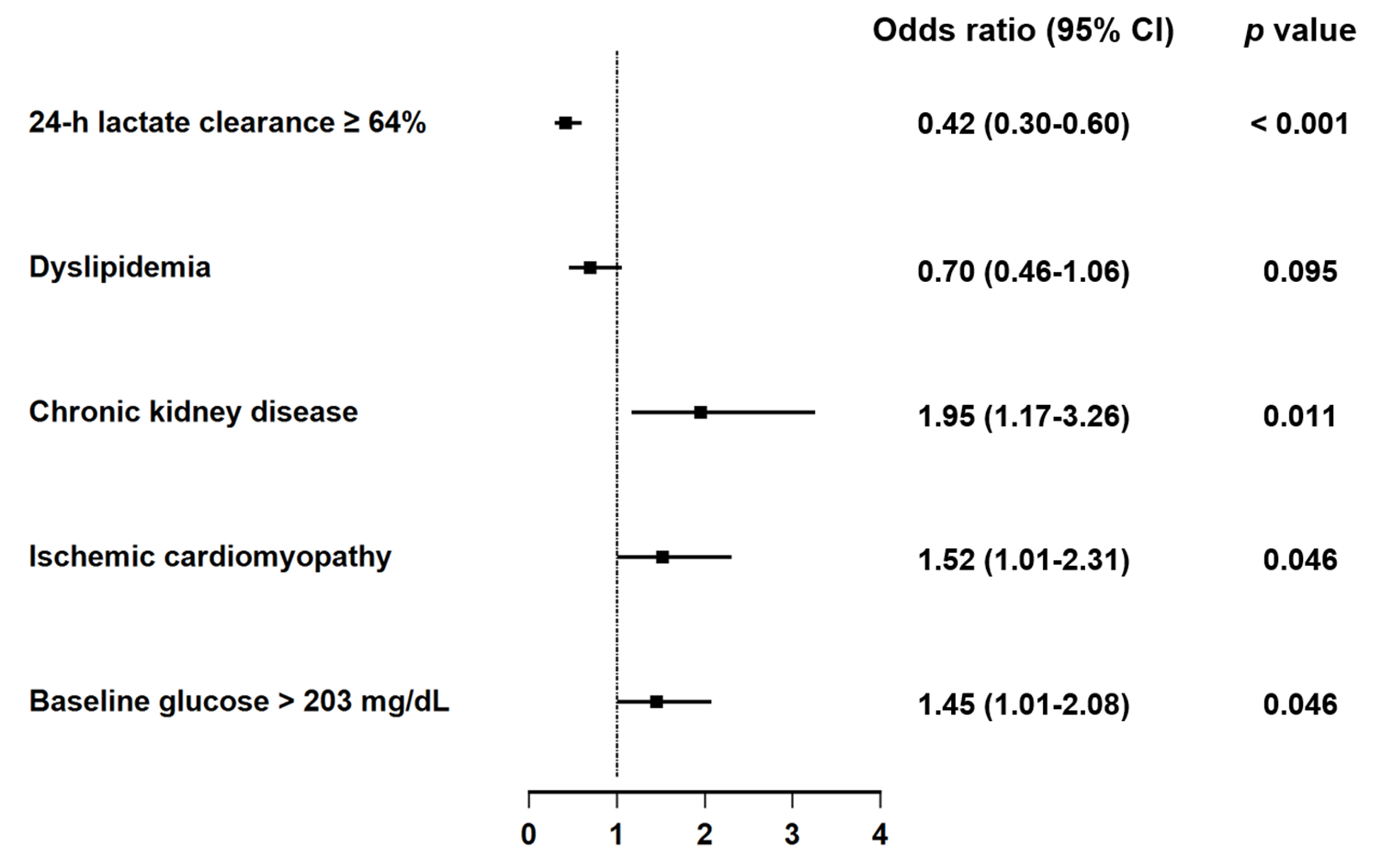

Supplement: Supplementary file 2 — Additional file 2: Figure S1. Predictors of in-hospital mortality. [file 40560_2021_571_MOESM2_ESM.docx]
