# Supplementary material for: Clinical significance of lactate clearance in patients with cardiogenic shock: results from the RESCUE registry
Source: J Intensive Care. 2021 Oct 18;9:63. doi: 10.1186/s40560-021-00571-7 (PMC8522140; doi:10.1186/s40560-021-00571-7)

**Additional file 3: Figure S2. ROC curves of lactate clearance to predict in-hospital mortality according to the application of mechanical circulatory support**

ROC curves show the comparison of 24-hour lactate clearance to predict in-hospital mortality according to mechanical circulatory support.

AUC = area under curve, ECMO = extracorporeal membrane oxygenation, IABP = intra-aortic balloon pump, ROC = receiver operating characteristic.


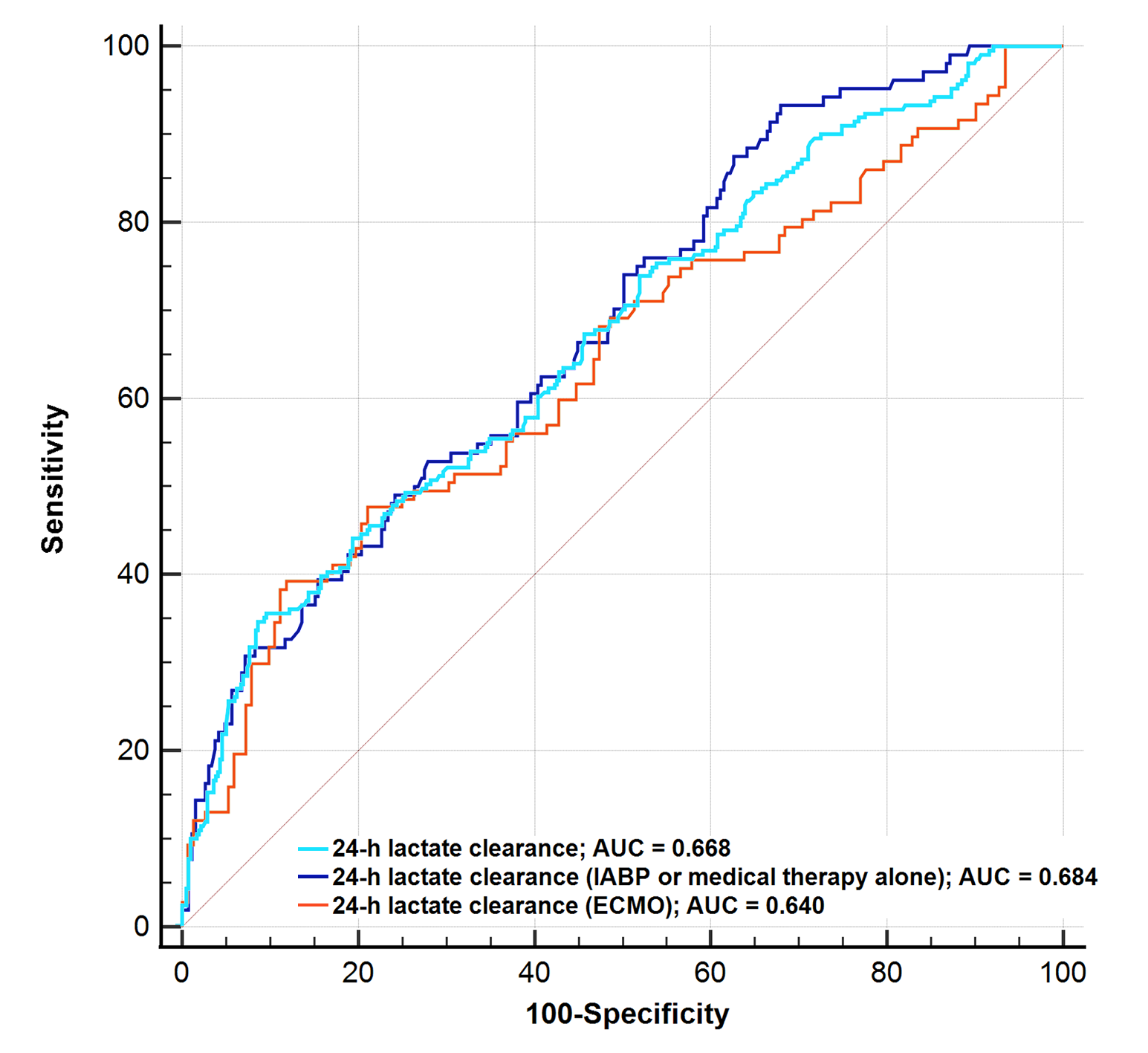

Supplement: Supplementary file 3 — Additional file 3: Figure S2. ROC curves of lactate clearance to predict in-hospital mortality according to the application of mechanical circulatory support. [file 40560_2021_571_MOESM3_ESM.docx]
